# Amino acid profiling in the gestational diabetes mellitus

**DOI:** 10.1186/s40200-016-0283-1

**Published:** 2017-03-31

**Authors:** Najmeh Rahimi, Farideh Razi, Ensieh Nasli-Esfahani, Mostafa Qorbani, Nooshin Shirzad, Bagher Larijani

**Affiliations:** 1 Endocrinology and Metabolism Research Center, Endocrinology and Metabolism Clinical Sciences Institute, Tehran University of Medical Sciences, Tehran, Iran; 2 Diabetes Research Center, Endocrinology and Metabolism Clinical Sciences Institute, Tehran University of Medical Sciences, Tehran, Iran; 3 Department of Community Medicine, Alborz University of Medical Sciences, Karaj, Iran

**Keywords:** Diabetes mellitus, Gestational diabetes, Amino acids

## Abstract

**Background:**

The prevalence of gestational diabetes mellitus (GDM) is increasing globally which is associated with various side effects for mothers and fetus. It seems that metabolomic profiling of the amino acids may be useful in early diagnosis of metabolic diseases. This study aimed to explore the association of the amino acids profiles with GDM.

**Methods:**

Eighty three pregnant women with gestational age ≥25 weeks were randomly selected among pregnant women referred to prenatal care clinic in Arash hospital of Tehran, Iran. Women divided into three groups including 1) 25 pregnant women with normal glucose tolerance test, 2) 27 pregnant women with diabetes type 2 (T2D) (n: 27) and 3) 31 women with GDM (n: 31). Plasma levels of amino acids were measured by high performance liquid chromatography and were compared in three groups. Statistical analysis was performed using SPSS 16.

**Results:**

Compared with normal mothers, GDM mothers showed higher plasma concentrations of Arginine (*P* = 0.01), Glycine (*P* = 0.01) and Methionine (*P* = 0.04), whereas the pregnant women with T2D had higher plasma levels of Asparagine (*P* = 0.01), Tyrosine (*P* < 0.01), Valine (*P* < 0.01), Phenylalanine (*P* < 0.01), Glutamine (*P* < 0.01) and Isolucine (*P* < 0.01). The results of regression analyses confirmed the significantly elevated in plasma concentration of Asparagine (OR:3.64, CI 1.22–10.47), Threonine (OR:3.38, CI 1.39–8.25), Aspartic acid (OR:3.92, CI 1.19–12.91), Phenylalanine (OR:2.66, CI 1.01–6.94), Glutamine (OR:2.53, CI 1.02–6.26) and Arginine (OR:1.96, CI 1.02–3.76) after adjustment for gestational age and BMI in GDM mothers compared to normal ones.

**Conclusions:**

Amino acids levels are associated with risk of GDM and diabetes mellitus. However further prospective studies are needed to clarify the role of different metabolites involved in mechanism of GDM.

## Background

Gestational diabetes mellitus (GDM) is a condition in which pregnant women without formerly diagnosed diabetes exhibit glucose intolerance in various degrees, especially during the third trimester [1]. The prevalence of GDM is increasing globally [2]. GDM is associated with many adverse neonatal and maternal outcomes such as increased risk of type 2 diabetes (T2D) and hypertension (HTN) which occurs in 2–9% of all pregnancies. The prevalence of GDM is estimated between 1 and 14% which varies depending on the studied population [3]. It is estimated that the increase in the prevalence of GDM will continue in coming years due to the ageing of population, urban sedentary lifestyle and the increasing number of obese women [4]. The worldwide prevalence of GDM varies by country. In fact, approximately one of every 20 pregnancies in Iran will be affected by complications of GDM [5, 6]. In a study conducted by Larijani et al., the prevalence of GDM was 4.7% (3.9–5.6%) in Tehran [7]. The prevalence of GDM in Iran has been estimated as 3.4% ranged from 1.3 to 18.6% [8]. Therefore, it can be well thought out that Iranian women are a particularly high- risk population.

GDM is associated with serious complications for both pregnant mother and her fetus [9]. There is a 2-fold increased risk of cesarean section and macrosomia, a 3-fold increased risk of neonatal hypoglycemia and hypocalcemia, and a 5-fold increased risk of still- birth in women with GDM compared with non-GDM ones [7]. Hence, the evaluation of the GDM should be considered at the first visit of pregnancy [10]. All acknowledged diagnostic methods used for screening of GDM are routinely carried out at 24–28 weeks of gestation based on monitoring of concentration of glucose in blood [11].

Metabolomic profiling, as a systematic study of small molecule products of biochemical pathways, is an efficient method for the identification of novel biomarkers and mechanisms related to a variety of diseases. The findings of previous metabolomic studies have indicated that branched chain amino acids (BCAAs), such as leucine, isoleucine, valine and aromatic amino acids, like tyrosine and phenylalanine could be used as a predictive marker of T2D [12].

Several studies have indicated that adiponectin, apolipoprotein CIII, and follistatin-like-3 can be considered as the early diagnostic biomarkers of GDM [13–15].

Recently, Bentley-Lewis et al. have maintained that the levels of six metabolites, including anthranilic acid, alanine, glutamate, creatinine, allantoin, and serine are significantly different between the GDM and control groups. In addition, the levels of BCAAs did not differ significantly between GDM and women with normal glucose tolerance [16].

However, the exact association between the concentration of amino acids in blood and the pathways of glucose metabolism in pregnancy is not yet fully elucidated. Therefore, we conducted this study to determine the plasma free amino acid profiling in women with GDM, diabetic pregnant and normal pregnant women to explore the blood profiles of amino acids and its association with GDM and diabetes mellitus during pregnancy.

## Methods

### Subjects

This case–control study was performed among pregnant women referred to prenatal care clinic in Arash hospital of Tehran, Iran, between Januarys to April 2016. Eighty three pregnant women aged 18–40 years with gestational age of ≥25 weeks were recruited. All participants were divided into three groups; 1) pregnant women with normal glucose tolerance test, 2) pregnant women with T2D before pregnancy or fasting blood sugar (FBS) > 95 mg/dl or 2-h post-GTT blood glucose <200 mg/dl in first trimester and 3) pregnant women with GDM based on ADA criteria [3].

The clinical exclusion criteria were: diabetes type 1, chronic complication of diabetes type 2 (nephropathy, retinopathy, and cardiovascular disease), gestational complication (premature delivery, premature rupture of membrane and pre eclampsia), chronic disease requiring medication during pregnancy, assisted reproductive technology pregnancies with the need to use hormones during pregnancy and renal diseases.

This study was approved by the Ethics Committee of the Endocrinology and Metabolism Research Institute (EMRI) affiliated with Tehran University of Medical Sciences. all participants signed an informed consent form and were informed about their rights according to the Helsinki declaration.

### Clinical and laboratory measurements

The questionnaires were completed by a trained nurse and the participants were requested to answer the questions carefully. Height and weight were measured according to a standard protocol by a team of trained nurses. Pre-pregnancy body mass index (BMI) was calculated using self-reported weight (kg) at 3 months before conception and divided by maternal height in meters squared (kg/m^2^). Brachial blood pressure was measured by trained nurses 3 times in the right arm after a 10-min rest period, using a manual mercury sphygmomanometer. A fasting blood sample was taken from all participants after 12 h fasting. Serum and plasma samples were separated from blood within 1 to 3 h following blood collection. Serum was separated by centrifugation at 500 × g for 10 min, and plasma was separated at 350 × g for 15 min at room temperature and then stored in −80 C until analysis. The concentration of FBS, urea and creatinine were measured by commercial kits (Pars Azmoon, Iran). Amino acid profiles were measured by HPLC method (Agilent Technologies).

### Statistical analysis

The Kolmogorov–Smirnov test was used to check the normal distribution of continuous variables. The continuous variables with and without normal distribution were described as means (standard deviations (SD)) and median (interquartile range (IQR)), respectively. Categorical variable were reported as number and percentages.

The plasma levels of amino acids between groups (NG, T2D and GDM) were compared using ANOVA for variables with a normal distribution and the Kruskal–Wallis test for variables with a skewed distribution. The multivariate multinomial logistic regression analysis applied to explore the association of each metabolite with the risk of developing GDM or T2D after adjustment for pregnancy age and BMI. Results of multinomial logistic regression analysis were reported as odds ratio (OR) and 95% confidence interval. Standardized scale (Z-score) of each metabolite were used in the multinomial logistic regression analysis. Statistical analysis was performed using SPSS 16. Considered-value less than 0.05 were considered as statistically significant.

## Results

The basic characteristics of the participants are presented in Table 1. As data show in Table 1, pregnant women with T2D had significantly higher BMI than controls (*P*-value = 0.03). As expected, we find that women with T2D or GDM had higher blood concentration of FBS than women with normal glucose tolerance (*P*-value <0.001). Maternal plasma amino acid concentrations in the 3 groups of pregnant women are presented in Table 2. Compared with normal mothers, GDM mothers show higher plasma concentrations of arginine (*p* value = 0.01), glycine (*p* value = 0.01) and methionine (*p* value = 0.04), whereas the pregnant women with T2D had higher plasma levels of asparagine (*p* value = 0.01), tyrosine (*p* value <0.01), valine (*p* value <0.01), phenylalanine (*p* value <0.01), glutamine (*p* value <0.01) and isoleucine (*p* value <0.01).

**Table 1:** General characteristics of pregnant women in three groups

Variables	NG (*n* = 25)	GDM (*n* = 31)	T2D (*n* = 27)	*P*-value
Age (year)	29.46 (5.45)	32.65 (5.56)	32.30 (4.89)	0.06
Weight (Kg)	72.63 (11.16)	81.34 (13.00)	82.92 (11.74)	**<0.001** ^**b, a**^
Height (m)	159.62 (3.63)	163.13 (6.70)	163.38 (7.05)	**0.04** ^**b, a**^
BMI (Kg/m^2^)	28.51 (3.66)	30.43 (3.50)	31.12 (4.51)	**0.03** ^**b, a**^
FBS (mg/dl)	78.75 (4.86)	96.44 (12.03)	146.09 (50.59)	**<0.001** ^**b, Ϯ**^
Pregnancy age (yrs)	28.48 (5.53)	29.14 (6.93)	20.29 (4.38)	**<0.001** ^**b, Ϯ**^
parity	3.04 (4.41)	2.34 (0.93)	2.04 (1.13)	0.38

^a^Comparison GDM and NG, ^b^comparison T2D and NG, ^Ϯ^Comparison GDM and T2D

Data are presented as mean (SD)

*NG* pregnant women with normal glucose tolerance test, *GDM* women with gestational diabetes mellitus, *T2D* pregnant women with type 2 diabetes mellitus

Boldface indicates significance of data with p_value <0.05

**Table 2:** Plasma concentration of amino acids in different groups of pregnant women

Amino acids (μUI/L)	GDM (*n* = 31)	T2D (*n* = 27)	NG (*n* = 25)	*P*-value
ASN	35.37 (28.23–40.53)	39 (22.09–53.69)	25.48 (19.70–31.81)	**0.01**
SER	67.54 (52.13–95.75)	65.44 (41.37–94.41)	72.85 (54.93–87.09)	0.60
HIS	39.57 (27.57–67.69)	33.88 (23.23–56.9)	45.78 (22.13–80.71)	0.39
ARG	61.50 (38.72–84.34)	34.45 (25.47–64.13)	41 (30.11–65.39)	**0.01**
CIT	20.62 (15.16–29.6)	23.02 (13.80–34.09)	16.69 (13.09–22.74)	0.26
GLY	135.48 (79.77–162.88)	44.04 (20.48–146.37)	105.51 (82.73–139.19)	**0.01**
THR	128.8 (86.79–206.44)	100 (64.56–197.83)	88.39 (69.38–133)	0.08
TYR	39.28 (30.70–46.64)	49.24 (40.1–66.6)	35.87 (25.58–42.13)	**<0.01**
MET	24.42 (17.56–29.01)	18.9 (14.19–25.45)	20.02 (14.59–23.07)	**0.04**
VAL	166.54 (129.21–210.76)	270.69 (215.06–366.65)	135.30 (110.36–175.52)	**<0.01**
PHE	49.74 (32.91–54.61)	54.26 (45.06–67.35)	42 (28.99–48.78)	**<0.01**
LEU	95.02 (67.14–1130.50)	104.38 (72.17–148.20)	73.52 (62.93–107.91)	0.07
ORN	21.55 (15.98–32.73)	19.51 (10.08–33.87)	19.7 (13.01–38.47)	0.50
ASP	11.01 (9.54–15.15)	10.22 (6.36–13.01)	9.16 (6.24–12.21)	0.05
GLU	98.60 (75.72–152.132)	124 (83.92–174)	71.41 (48.13–114)	**<0.01**
GLN^c^	312.86 (89.18)	267.42 (86.9)	298.59 (123.41)	0.20
ALA^c^	288.47 (97.03)	271.77 (119.82)	290.52 (133.26)	0.80
TRP^c^	32.97 (13.15)	35.4 (18.89)	36.08 (19.94)	0.76
ILE^c^	55.38 (18.94)	84.28 (34.53)	52.01 (20.91)	**<.001** ^**Ϯ, b**^
LYS^c^	175.44 (58.35)	134.59 (65.11)	164.38 (85.24)	0.07

^a^Comparison GDM and NG, ^b^comparison T2D and NG, ^Ϯ^Comparison GDM and T2D

^c^Data presented as mean (SD)

Data presented as median (IQR)

According to Kruskal Walis

Boldface indicates significance of data with p_value <0.05

The results of multivariate multinomial logistic regression indicated that the increase in plasma level of amino acids, including asparagine (OR: 3.64, 95% CI: 1.22–10.74), threonine (OR: 1.95, 95% CI: 1.05–3.60), aspartate (OR: 3.92, 95% CI: 1.19–12.91), phenylalanine (OR: 2.66, 95% CI: 1.01–6.94), glutamine (OR: 2.53, 95% CI: 1.02–6.26) and arginine (OR: 1.83, 95% CI: 1.02–3.30) increased the risk of GDM (Table 3). In addition, an increase of one SD of plasma level of amino acids such as asparagine (OR: 2.36, 95% CI: 1.15–4.65), isoleucine (OR: 4.81, 95% CI: 2.10–11.03), valine (OR:7.70, 95% CI: 2.66–22.27), phenylalanine (OR: 3.01, 95% CI: 1.21–6.26) and glutamine (OR: 2.99, 95% CI: 1.26–7.11) increased the risk of T2D compared to normal pregnant women (*P*-value <0.05 for all).

**Table 3:** Association of amino acids with GDM, T2D and normal pregnancy in nominal logistic regression

Amino acid		Crude			Adjusted^a^		
		OR	95% CI	*P* value	OR	CI	*P* value
ASN	GDM/NG^a^	1.57	0.77–3.22	0.21	3.64	1.22–10.74	**0.01**
	T2D/NG	2.36	1.15–4.85	**0.01**	6.78	1.92–23.91	**<0.01**
SER	GDM/NG	1.10	0.66–1.83	0.70	1.25	0.67–2.34	0.46
	T2D/NG	0.79	0.42–1.46	0.45	0.73	0.32–1.63	0.44
HIS	GDM/NG	0.68	0.39–1.16	0.15	0.74	0.41–1.33	0.32
	T2D/NG	0.46	0.23–0.89	**0.02**	0.27	0.08–0.84	**0.02**
GLN	GDM/NG	1.25	0.73–2.14	0.40	1.39	0.75–2.57	0.29
	T2D/NG	0.65	0.34–1.21	0.18	0.73	0.34–1.57	0.42
CIT	GDM/NG	1.10	0.6–2.03	0.74	1.40	0.60–3.23	0.43
	T2D/NG	1.84	0.90–3.74	0.09	1.44	0.65–3.17	0.36
GLY	GDM/NG	1.03	0.62–1.71	0.32	0.75	0.43–1.31	0.31
	T2D/NG	0.48	0.25–0.94	**0.03**	0.41	0.16–1.05	0.06
THR	GDM/NG	1.95	1.05–3.60	**0.03**	3.38	1.39–8.25	**<0.01**
	T2D/NG	1.52	0.77–2.98	0.22	4.12	1.49–11.41	**<0.01**
ILE	GDM/NG	1.24	0.59–2.57	0.56	1.19	0.54–2.62	0.69
	T2D/NG	4.81	2.10–11.03	**<0.01**	4.99	1.63–15.20	**<0.01**
LEU	GDM/NG	1.36	0.71–2.61	0.30	1.50	0.67–3.36	0.32
	T2D/NG	2.32	1.15–4.68	**0.02**	1.35	0.56–3.25	0.51
ORN	GDM/NG	0.79	0.46–1.34	0.37	0.86	0.47–1.55	0.62
	T2D/NG	0.76	0.42–1.35	0.34	0.38	0.15–0.97	**0.01**
LYS	GDM/NG	1.18	0.66–2.11	0.57	1.05	0.95–1.15	0.29
	T2D/NG	0.58	0.29–1.14	0.12	0.83	0.76–0.93	**<0.01**
ASP	GDM/NG	2.44	0.90–6.65	0.08	3.92	1.19–12.91	**0.02**
	T2D/NG	2.31	0.83–6.42	0.11	3.19	0.95–1.067	0.06
TYR	GDM/NG	1.09	0.54–2.21	0.79	1.08	0.51–2.26	0.83
	T2D/NG	2.64	1.27–5.50	**<0.01**	1.74	0.76–3.96	0.19
TRP	GDM/NG	0.82	0.48–1.42	0.42	0.79	0.45–1.40	0.43
	T2D/NG	0.96	0.56–1.65	0.89	0.64	0.29–1.42	0.27
MET	GDM/NG	2.48	0.64–9.57	0.18	4.57	0.94–21.91	0.05
	T2D/NG	0.81	0.45–1.45	0.48	0.77	0.36–1.65	0.50
VAL	GDM/NG	1.89	0.71–4.51	0.21	2.11	0.77–5.77	0.14
	T2D/NG	7.70	2.66–22.27	**<0.01**	4.85	1.55–15.14	**<0.01**
PHE	GDM/NG	2.07	0.95–4.55	0.06	2.66	1.01–6.94	**0.04**
	T2D/NG	3.01	1.21–6.26	**<0.01**	3.12	1.09–8.95	**0.03**
GLU	GDM/NG	2.19	0.93–5.14	0.07	2.53	1.02–6.26	**0.04**
	T2D/NG	2.99	1.26–7.11	**0.01**	3.26	1.19–8.88	**0.02**
ARG	GDM/NG	1.83	1.02–3.30	**0.04**	1.96	1.02–3.76	**0.04**
	T2D/NG	0.84	0.44–1.59	0.59	1.00	0.46–2.18	0.90
AlA	GDM/NG	1.20	0.71–2.04	0.48	1.42	0.76–2.65	0.27
	T2D/NG	0.84	0.47–1.50	0.56	0.90	0.41–1.99	0.81

*NG* pregnant women with normal glucose tolerance test, *GDM* women with gestational diabetes mellitus, *T2D* pregnant women with type 2 diabetes mellitus

Metabolite levels were standardized to SD unit

^a^adjusted for gestational age and body mass index (BMI)

Boldface indicates significance of data with p_value <0.05

## Discussion

To our knowledge, this is the first report describing the effect of GDM and T2D on the concentration of amino acids during the pregnancy period among Iranian women. We conducted an exploratory metabolomic analysis on 83 pregnant women including 31 women with GDM, 27 women with T2D and 25 women with normal glucose tolerance. The highest plasma levels of arginine, glycine, and methionine were observed in GDM women, while the pregnant women with T2D had the highest plasma level of asparagine, tyrosine, valine, phenylalanine, glutamic acid, and isoleucine compared to other groups which were statistically significant. In the present study, decreased plasma levels of serine, histidine, alanine, and tryptophan were also observed in both groups of pregnant women with GDM or T2D. However, these differences were non-significant.

There are limited studies to date evaluating the variations in the plasma levels of amino acids in GDM. Cetin et al. in their study of 16 normal and 17 GDM pregnancies have shown that only ornithine significantly increased in GDM mothers. Also, An increase in the levels of valine, methionine, phenylalanine, isoleucine, leucine, ornithine, glutamate, proline, and alanine along with a significant decrease in the level of glutamine in umbilical vein and artery of women with GDM were reported compared to the normal pregnant women [17]. However, it is important to underline the deference between the 2 studies. The BMI of GDM mothers in our study was higher than those in Cetin et al. study (30.43 ± 3.50 vs. 28.0 ± 1.6 kg/m^2^). The results of our study illustrated a significant elevated plasma level of Asparagine, Threonine, Aspartate, phenylalanine, glutamate, and arginine in GDM women after adjustment for gestational age and BMI.

In the previous studies, the targeted metabolimoic assays have demonstrated the increased level of alanine, proline, glutamine/glutamate, arginine, and asparagine/aspartate in subjects with higher level of fasting blood sugar [18]. Bentley-Lewi et al., by conducting a nested case–control study among pregnant women aged 18–40 year, reported that of the 91 studied metabolites, one can name anthranilic acid, alanine, glutamate, allantoin, and serine as being significantly higher and creatinine as significantly lower in GDM women compared to non-GDM ones [16].

Previous studies have confirmed that BCAAs, including isoleucine, leucine, and valineas well as aromatic amino acids such as phenylalanine and tyrosine were strongly associated with insulin resistance and T2D [12, 19–22]. However, there is a controversy about the association between BCAAs and GDM. Although some researchers have found elevated concentration of branched amino acids in blood of those suffering GDM [23], other studies have not confirmed this association [17, 24]. In consistent with the increased of BCAAs in type 2 diabetic patients, the results of the present study showed higher plasma concentration of valine, isoleucine in T2D pregnant women compared to GDM and non-GDM ones. It was shown that an increase in the hepatic gluconeogenes entails the increased level of BCCAs in blood. Patients with insulin resistance have reduced activities of BCAAs catabolic enzymes in liver and adipose tissues, which are associated with the higher blood concentration of BCAAs [19, 25]. Some of the previous studies have demonstrated that aromatic amino acid are associated with insulin resistance [26] especially in pregnant women with T2D [12] and GDM [27] while, other investigations didn’t find any statistically significant correlation between the plasma level of these amino acids and GDM [17, 23, 24]. In present study, we found significantly elevated levels of tyrosine and phenylalanine in T2D, but not among GDM mothers.

Another study found that of the 21 amino acids, only methionine, glycine, alanine, citrulline, and ornithine levels were significantly higher in normal pregnant women compared to those with GDM [28]. Similar to this study, only the plasma level of alanine was higher in normal pregnant women compared to GDM and T2D groups which was statistically non-significant. The mechanisms of these observations were investigated in the same study. They suggested that the elevation of ketone bodies in the GDM group may inhibit the proteinolysis and reduces the oxidation of BCAAs and other ketogenic amino acids in skeletal muscles, simultaneously. Therefore, the ketogenic amino acids (lysine) and the branched-chain amino acids, such as isoleucine and leucine are released at low rates from skeletal muscle and are mostly catabolized in the liver rather than in peripheral tissues in GDM mothers [28].

Analyses of conventional metabolites of 67 Northern European mothers with high FBS (>90th percentile) and 50 mothers with low FBS (<10th percentile) indicated that high FBS mothers had higher blood level of alanine, proline, and BCAAs that together with higher triglycerides, 3-hydroxybutyrate, had consistency with insulin resistance in this groups [29]. In this study, the metabolic profiling were determined using targeted and untargeted metabolomic assays. However, in contrast to this study, we observed that the plasma level of alanine was higher in normal pregnant women compared to those with GDM or T2D. The elevated levels of valine, and isoleucine in pregnant women with T2D compared to other groups confirm the association of BCAAs and hyperglycemia in this study and the previous ones [30–32].

The controversy in these results could be explained by the differences in the genetic background and environmental influences on GDM group. Previous studies have been implemented through using different methods to assess metabolomic profiling, including targeted and untargeted approaches to metabolomic using ultra-performance liquid chromatography tandem mass-spectrometry (UPLC-MS) or high-resolution gas chromatography time-of-flight mass spectrometry or liquid chromatography tandem mass spectrometry (LC-MS/MS) and etc. for targeted and untargeted. In this study, ultra-performance liquid chromatography was used for the screening of metaboloms in plasma samples. In addition, differences in the efficient control of glucose and the trimesters of pregnancy may be other reasons.

The limitation of the present study is the small sample size. Therefore, the cohort studies with larger sample sizes are needed to investigate the role of metaboloms including free amino acids in the prediction of GDM.

## Conclusion

In conclusion, this investigation showed that amino acids levels are associated with higher risk of T2D and GDM. However, further prospective studies should be conducted to clarify the role of different metabolites involved in the mechanism of GDM. By considering the increasing prevalence of diabetes in the world, effective methods such as metabolomic investigations are needed to make the prediction and early diagnosis of GDM possible in pregnant women.
